# Understanding Young People and Their Care Providers’ Perceptions and Experiences of Integrated Care Within a Tertiary Paediatric Hospital Setting, Using Interpretive Phenomenological Analysis

**DOI:** 10.5334/ijic.5545

**Published:** 2020-10-27

**Authors:** Hannah Johnson, Megan Simons, Dana Newcomb, Erika Borkoles

**Affiliations:** 1 Integrated Care, Children’s Health Queensland Hospital and Health Service, Level 9, Centre for Children’s Health Research, South Brisbane QLD, AU; 2 Department of Occupational Therapy, Queensland Children’s Hospital, South Brisbane QLD, AU; 3 Centre for Children’s Burns and Trauma Research, Child Health Research Centre, Level 7, Centre for Children’s Health Research, South Brisbane QLD, AU; 4 Primary Care Clinical Unit, Faculty of Medicine, University of Queensland, AU; 5 School of Medicine, Public Health, Griffith University, Gold Coast, QLD, AU

**Keywords:** integrated care, patient experience, family-centred care, paediatrics, healthcare providers, person-centred care

## Abstract

**Introduction:**

Benefits of integrated care include improved health outcomes and more satisfaction with experiences of care for consumers. For children and young people with chronic and complex health conditions, their care may be fragmented due to the multitude of healthcare providers involved. This paper describes the experiences of integrated care in a paediatric tertiary hospital.

**Theory and methods:**

Using an Interpretive Phenomenological Analysis approach, semi-structured interviews were conducted with children and young people, their parents and healthcare providers to explore stakeholders’ integrated care experiences.

**Results:**

Nineteen interviews were completed (6 children and young people, 7 parents and 6 healthcare providers) and transcribed verbatim. Two recurrent themes were applicable across the three cohorts: ‘agency and empowerment’ and ‘impact of organisational systems, supports and structures’.

**Discussion and conclusion:**

Stakeholders’ experiences of integrated care highlighted the need to examine the discrepancies between healthcare strategies, policies and service delivery within a complex, and often inflexible organisational structure. Power imbalance and family agency (including directly with children and young people) needs to be addressed to support the implementation of integrated care.

## Introduction

Healthcare for children with complex and chronic medical conditions is often fragmented as a result of high healthcare utilisation and frequent hospitalisation [1]. Consequently, these children are more susceptible to preventable medical errors, higher risk of hospital readmission and variation in healthcare [1,2,3,4,5,6]. Evidence from qualitative interviews has highlighted that children and young people with complex health needs require care that is integrated (both within and between primary, secondary and tertiary healthcare providers and other non-health sectors, e.g. education), and holistic and family-centred [7,8]. Parents often report heightened levels of carer burden as a result of attempting to navigate ‘the system’ due to lack of assistance in care coordination, communication and/or the necessary education to navigate both child and family care needs [1,3,5].

Integrated care can improve health outcomes and experiences of care for children and young people with chronic and complex conditions, as well as improve the efficiency of services, and improve staff satisfaction [1,7,9,10,11]. There are many definitions of integrated care [12] which may refer to models of care or interventions such as care coordination, case management, co-location of multidisciplinary teams; commissioned or jointly funded programs; or specific working approaches [13]. For clarity, in this study, we adopt the Children’s Health Queensland Hospital and Health Service (CHQHHS) definition, which is based on Ziniel et al’s [14]:

“Integrated care is the provision of care in the broadest sense – physical, psychological and social – which is oriented around the needs of children, young people and families, and designed and delivered in partnership with them. In an integrated system, these needs are met through the coordinated and collaborative working of all providers, irrespective of sectorial, organisational or geographic boundaries.” [15, p. 5]

CHQHHS’s definition incorporates person-centredness as a central component of integrated care, recognising that a person and their immediate family and/or carers must be empowered and engaged within the healthcare system and their own care coordination [15,16,17].

Health systems typically rely on parents to provide their child’s (the patient’s) perspective, however children and young people have the right to express their own viewpoint [18]. Collecting lived experience data directly from children and young people provides credible and valid information that can allow us to gain insight into their wellbeing, perceptions of outcomes and experiences [19]. It is important to also seek the parents’ views in their own right, not just as a child’s proxy, as parents have expertise in the everyday care needs of their children, are integral to decision making and participation in their child’s healthcare, and are often the first point of contact for the patient [8,20,21,22]. The power of collecting both parental and child lived experiences means that each lived experience can be examined in their own right and can drive meaningful health service design. It is equally important to understand the challenges healthcare providers face when delivering and coordinating their care, from both an individual and organisational point of view [1,2,13,21]. There are cultural, hierarchical, behavioural, environmental, and attitudinal barriers to why more of a shift to integrated care hasn’t been made in many hospitals and healthcare systems to date [23]. By working with families and healthcare providers directly to explore these barriers, engagement can be fostered to co-create change and determine a way forward, with ‘buy-in’ for any changes required [10]. To date, there is no published literature to our knowledge that explores the experiences of integrated paediatric tertiary care, from all three perspectives, that is, children and young people with complex health needs, their parents and their healthcare providers. Therefore, this study was framed by a main research question: How do children and young people with complex and chronic conditions experience integrated care at a tertiary paediatric hospital? The focus was specifically on exploring children’s and young people’s, their parents’ and healthcare providers’ lived experiences of care, their perception of barriers and enablers of integrated person-centred care, and what they believe are the opportunities to improve integration of care in a tertiary paediatric hospital.

## Method

### Research design

An Interpretive Phenomenological Analysis (IPA) approach was selected for this study as it recognises how important context is and how a person makes sense of a situation or experience in that context [24]. The IPA approach is idiographic, meaning that researchers conduct detailed examination of individual cases, to understand each situation, resulting in recommended sample sizes of four to ten [24]. This provides opportunity to analyse the similarities and differences between cases thoroughly to derive meaning from experiences.

The Standards for Reporting Qualitative Research (SRQR) were used to guide the preparation of this research and manuscript [25].

The project team had varied backgrounds, including public health (HJ), integrated care (HJ, DN), primary care (DN), occupational therapy (MS), psychology (EB) and implementation science (EB, MS).

Ethical and governance approval for this research was obtained from the Children’s Health Queensland Hospital and Health Service Human Research Ethics Committee (HREC reference number: LNR/2018/QCHQ/44246). Administrative ethical approval was also obtained through Queensland University of Technology (QUT approval number: 1800000900).

### Study population and setting

Purposive sampling of three cohorts of participants (children and young people, parents, and healthcare providers) who share a lived experience of chronic and complex healthcare needs living within the greater Brisbane region was used to enhance transferability [24]. Children and young people with chronic and complex healthcare needs are also known as children with medical complexity (CMC) and are defined as: “children and youth with serious chronic conditions, substantial functional limitations, increased health and other service needs, and increased healthcare costs” [6, p. 203]. The participants were recruited through their engagement with the Connected Care Program (CCP), a nurse-led program for children and young people who see multiple specialist teams at the Queensland Children’s Hospital (QCH), Brisbane, Australia established in 2013. Eligibility for CCP are children and young people aged <16 years for new patients, or <18 years for continuing patients until discharged from paediatric care. Criteria is based on chronicity, complexity, fragility, and intensity of care needs. Once on the program, the CCP nurses coordinate children and young people’s access to healthcare providers and support services both at QCH and within regional health services closer to the child’s home, across geographical, sectorial and organisational boundaries. They provide a single point of contact for families and aim to coordinate the care around the family’s needs. By improving communication and linkages between service providers, the coordinators ensure a child’s care is managed seamlessly across acute, community and primary healthcare sectors.

The children and young people participants in the research were patients enrolled in the CCP, and the healthcare provider participants were purposively and conveniently invited to participate if they were healthcare providers of the children and young people included in the study. The authors are not aligned with the CCP and first author (HJ) is non-clinical member of staff. The QCH is the only paediatric tertiary hospital in Queensland, Australia.

### Data collection

The qualitative in-depth, semi-structured interviews completed by the first author (HJ) were an opportunity for participants to tell their own story, using their own words [24,26]. An interview schedule was developed with open-ended questions and areas for exploration with each cohort. This can be seen in supplementary file 1. The interviews were conducted at a time convenient to each the participants (an interview per family) from October 2018 to March 2019. The majority of interviews were conducted in a meeting room within the QCH campus, with one parent interview conducted via telephone. The children/young people and parents were interviewed while the other remained in the room. This was due to the age and developmental stage of the child or young person and limitations for supervision of the child/young person if not with their parent. Parents were asked to allow their child to respond to the interview questions as they wished without interference. Informed written and verbal consent was obtained from all participants before commencing the interview and all interviews were audio-recorded.

### Data analysis

**Figure 1:**
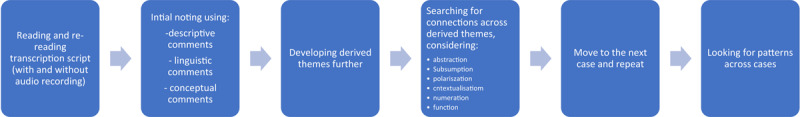
IPA data analysis process.

Analysis by the authors followed the IPA guidelines [24]. Interviews were transcribed verbatim and de-identified. Interpretive thematic analysis [26] was completed by a single coder (HJ), with 20% coded by a second coder (MS) [24]. Recurring meetings (HJ, EB, MS, DN) were held throughout the analysis phase for iterative discussion, to check meaning and discuss emerging themes until consensus. This process also enhanced credibility and confirmability of the data, reduced researchers’ bias based on their fore-conceptions (prior experiences, assumptions and preconceptions) and to ensure appropriate cyclical interpretation (hermeneutic circle) of the phenomena was achieved [24]. Each transcript was analysed individually to ensure each case was appreciated on its own terms, to respect its individuality, allowing for different themes to present themselves if relevant as per the IPA approach (Figure 1). All derived themes (at individual level) were compared for convergence and divergence. Converging themes were identified within and across participant cohorts to present a final list of superordinate themes.

## Results

Nineteen participants were recruited. Six interviews were completed with children and young people (16% male) aged between 7 and 15 years (M = 11 years, SD = 3 years), lasting between 10 minutes and 21 minutes (M = 15 minutes, SD = 4 minutes). The children and young people interviewed all had mild intellectual impairment, which impacted how the experiences were interpreted. Each child and/or young person was engaged with multiple healthcare services/teams and their condition(s) affected multiple body systems.

Six interviews were conducted with the children and young people’s parents (43% male), lasting between 43 minutes and 99 minutes (M = 63 minutes, SD = 24 minutes). Five interviews were with a single parent, and one was conducted with both parents (mother and father). The families lived an average of 20kms away from QCH (range 7 kms to 38 kms).

Supplementary file 2 provides further details on the demographics of the participating families.

The healthcare providers interviewed included two CCP nurses, an occupational therapist, a social worker, and a medical professional, all based at QCH, and one community-based general practitioner. The 6 interviews with healthcare providers lasted between 38 minutes and 78 minutes (M = 60 minutes, SD = 14 minutes). To maintain confidentiality of participants, the terms ‘Medical professional’, ‘Allied health professional’ and ‘Nurse’ have been used.

The IPA approach requires the researchers to analyse each case individually, then as a cohort to identify the recurrent themes. There were 14 superordinate themes identified between the three cohorts (children and young people, parents, and healthcare providers), detailed in supplementary files 3, 4 and 5, respectively. Following this, two recurrent themes were derived across the three cohorts which were ‘agency and empowerment’ and the ‘impact of organisational systems, supports and structures’.

### Agency and empowerment

Agency can be described as the ability of a person to influence or control what happens to them.

All parents felt they and their children had been disempowered by some healthcare providers and by ‘the system’. Both parent and healthcare providers experienced and described the power imbalance between the healthcare providers and families that impacted the care the child received and the parents’ engagement with the system.

“They sort of see us as, I guess, uneducated. We don’t have a medical degree and I totally understand that, however we know our daughter better than anybody else.” (Parent 2)

“¨sometimes it’s power and sometimes it’s the belief that because – and I’m saying we, generically, the belief that just because we are at the Queensland Children’s Hospital and we are the senior person in that clinic, it does not mean we know more than the family.” (Nurse 1)

Some healthcare providers acknowledged that the way information is delivered to families does not encourage families to fully participate in decisions about care or understand what is going on for their child.

“But I think other clinicians might have a very standard way of delivering information, and not necessarily adjust it or check in with the patient that they’ve understood and that they actually are free to ask questions.” (Medical professional 1)

Evidently this lack of agency (i.e., the ability of a person to influence or control what happens to them) and power struggle caused anxiety, stress and in some instances, frustration and a feeling of disassociation and trauma from poor outcomes and adverse events.

“I’d like my voice to be heard because this was fairly traumatic for us.” (Parent 3)

The healthcare providers reflected that other clinicians often do not recognise that the family are an ‘expert’ in their child. If they were empowered and supported, the partnership between healthcare provider and patient and family would deliver better outcomes for all involved.

“¨we need to empower them a little bit more¨” (Nurse 2)

Going to hospital caused anxiety for all children and young people interviewed, leading to a feeling of not wanting to be there. This was reflected through fear of pain and/or fear of medical errors due to previous poor experiences or outcomes, manifesting as a lack of agency and power in their own care.

“***Interviewee:*** I feel a bit anxious and worried what’s going to happen.

***Interviewer:*** Sure. Why do you think you feel like that?

***Interviewee:*** Because I don’t know when something is happening and I don’t know if it were to happen, the doctors and the nurses would tell me.” (Young person 1, 15 years)

Many children and young people felt they weren’t included in discussions about their care or making choices about their care.

“They just talk to mum and dad.” (Child 3, 7 years)

“Yeah, I don’t get to choose what happens.” (Child 1, 10 years)

Some children and young people could explain how they felt though could not identify the reason for the way they were feeling. The children were unable to express their emotions in the context of care because they felt they didn’t have agency, that is, people often didn’t listen to them. The children and young people recognised that they must participate in their care because healthcare providers and their parents want them to, so they resigned themselves to go through the motions just to get it done, rather than being fully involved.

“I don’t really know why but because I had to – I don’t really know why.” (Child 1, 10 years)

All parents felt they had to continually advocate for their child, but many often found that they needed healthcare providers to do so on their behalf to be taken seriously.

“¨as the years have gone on and I’ve had to advocate so many times, that I’ve had to come out of my shell because I’ve had to stand up for my daughter. It’s been a bit frustrating because I don’t think it really should have been my place.” (Parent 3)

It was recognised by parents and healthcare providers that the CCP model provided advocacy for the family, whilst also empowering the family, thereby building their agency over time.

“¨what a game-changer that [CCP] was, because it meant we had someone who knew us and listened and knew – before we’d go in – what our concerns, questions were, and made sure that we had answers to those.” (Parent 4)

“¨ [CCP] one person to contact all the time who can help coordinate some of their appointments, but not only that, can actually offer them some further detail and information around understanding the medical jargon that doctors use, the need for certain tests and appointments but also bring a whole big support about when they are actually at home and living in their own community that they can actually tap into other resources there as well.” (Allied health professional 2)

The role the CCP nurses played in providing medical advocacy appeared additional, but highly valued, to the core responsibility of the CCP model (i.e., coordinating care, providing a single point of contact, and to be a conduit between family and health system). These supports, along with the advocate-role, were described positively across all parent experiences, and healthcare providers echoed this value.

“In talking about integrated care, that would not have happened without Connected Care [CCP] helping to liaise between all the different teams and making that happen smoothly and efficiently, but also connecting back to us, and communicating with us and taking that stress away from us – reducing it, not taking it away, but definitely reducing. Things like that, you can’t put a value on.” (Parent 4)

“¨they’re that kind of one reliable contact point for the parents at the hospital when they have multiple healthcare providers within that same hospital and they can provide that communication link as well as organising the logistical issues with appointments and that kind of thing” (Medical professional 1)

Collectively healthcare providers’ and parents’ lived experiences indicated that the default delivery mechanism of specialist tertiary care was experienced in a siloed and fragmented way, despite these families being part of the CCP program. It appeared that the traditional ‘medical model’ of providing care was still a common practice, and the associated mentality and practice of this was a significant barrier to delivering integrated care more broadly. Healthcare providers and parents described that many healthcare providers continue to only look after their own speciality when treating the children and young people interviewed in this study. The holistic needs of the children and young people and needs of their family units, their social determinants of health, such the continuity of their education was also inconsistently addressed.

“The clinical specialties very much operate from a clinical specialty and you have families who – we don’t take into account family life¨” (Nurse 1)

“I feel that hospitals really work in a very – well we know the medical model. That certainly doesn’t always suit the health and wellbeing of children, necessarily.” (Allied health professional 1)

The parents were relied upon to ‘join the dots’, within an organisation and system that they lacked expert knowledge and understanding of. This was perceived differently to coordinating care in terms of what CCP provided.

“[Speciality 1] will say, deny – oh, this isn’t our area it must be a [Speciality 2] problem. [Speciality 2] will say, no that’s not our issue either, it’s a [Speciality 1] problem. So, they obviously don’t like coming together to find a solution. They just want to say, nope that’s not me… As a parent, as an – I don’t have a doctorate, but you know what? I can see that there is a middle ground. It’s a body and there needs to be someone who will look after this area and there is no one. There is no one who looks after the [body part]. So, let’s find someone who will. Let’s have someone put their hand up and go, you know what, I can work with you and together we can solve this. There isn’t any of that.” (Parent 2) (the actual specialities and body part mentioned were removed for confidentiality reasons)

Positive experiences of care were described from each parent when they felt like they were a partner in their child’s care. This was when communication with them and their child, and between healthcare providers was consistent, respectful, open and provided at a level and time that suited the family’s needs and successfully managed the parents’ expectations and which also respectfully involved them in care decision making of their child.

“¨they just come up to me and they talk about hospital and I really like them very much because they are very kind.” (Child 4, 10 years)

“That’s what family-centred care looks like. You share information. You have respect. You go, I’m going to respect – again, I respect this parent and I’m going to treat them with dignity. I’m going to – we’re sharing information equally – back and forth – being open and transparent. We’re partners and full participation. I didn’t feel like a second-class citizen. I was – I had a seat at all the meetings.” (Parent 4)

However, communication between healthcare providers and families was experienced as inconsistent, but both cohorts recognised this and wanted to improve integration of care and were frustrated by providing and receiving a poor service.

“¨it’s poor service delivery to not have that communication as being just as important – as an important part of what you provide, care wise, as the actual care or treatment recommendations or whatever” (Medical professional 1)

### Impact of organisational systems, supports and structures

Organisational systems, supports and structures influenced how children and young people and their parents experienced care, as well as either hindered or supported healthcare providers’ ability to deliver integrated care. For children and young people who see multiple teams and healthcare providers, there are many stakeholders to keep informed of care plans, interventions and treatment, and for the families to visit at the hospital for consultations. Some parents and healthcare providers described the scheduling of clinics as disconnected, particularly before parents had the CCP nurse to assist with coordinating appointments. This affected how much the hospital and the child’s condition impacted the family’s life.

“Yeah, it’s definitely an area that without them [CCP] the hospital just isn’t consistent enough and there are certain clinics that are worse for it, but I think they all need to just have one way of doing it as a hospital¨” (Parent 2)

Healthcare providers reflected on the complexity of staffing, resourcing, and scheduling and rostering of staff and clinics.

“¨it’s difficult because you’ve got clinics being run at different times, and at different frequencies.” (Medical professional 1)

They recognised the impact on parents.

“All the other medical people are rotating and changing, and that’s the bit that makes it confusing for families because they don’t understand why they haven’t seen their doctor and they see this other doctor and they don’t understand me or my doctor keeps coming but who are these other people?” (Allied health professional 2)

Poor communication caused much angst and frustration amongst parents and healthcare providers. For parents, their main experience was inconsistency of care and practice amongst healthcare providers, and healthcare providers echoed this.

“I’d say there are so many great people, but it depends which day, which team – and which team on which day – that you potentially get.” (Parent 4)

“How is there such a big difference in the level of care, just because it’s a different person? When – is the hospital meant to regulate that? Is it – how do they know that the level of care is so different between different doctors in the same specialty?” (Parent 2)

The importance of care coordination as a support system for parents was again highlighted by parents and healthcare providers, to deal with the complexity of the scheduling and staffing systems, and also as a key facilitator for good communication.

“…just having that one person they need to contact and get things organised just reduces their stress significantly.” (Medical professional 1)

Some parents described the quality of communication and care varied according to the particular provider they saw that day and how this affected their experiences of the hospital.

“The thought of going back to hospital makes me feel sick. That’s how hard it is, because again, you don’t know which [specialist] you’re going to get, which pre-op nurse. All those conversations – are they going to listen to you about what works well for [child] and how terrified she is?” (Parent 4)

For the children and young people, the impact of the systems and supports were not easy to describe. They did however, describe what happened to them, and what they experienced at hospital, particularly interventions such as blood tests, x-rays, and physical examinations, and how the healthcare providers and the integrated services interacted with them. Interventions that caused the least anxiety included x-rays and physical examinations, reportedly due to opportunities for distraction and provision of reward (stickers).

“I love the MRI and CT scan ever since last time I did that I was very still and I’m so afraid of noises and I ignored the noises, I watched the movie¨” (Child 4, 10 years)

Other aspects of care the children and young people had a positive experience with included people being kind to them, watching television, playing with toys, drawing, or having their parents beside them at point of care. This could reflect the structure of normal childhood, as these activities may help them feel closer to what they are missing out on by being at hospital.

“I was so pleased to have my parents back and staying and sleeping with me¨” (Child 4, 10 years)

Both healthcare providers and some parent participants reflected on values. Individual and organisational values were seen as important to guide and empower healthcare providers. However, culturally it was reported that these did not appear to reflect all healthcare providers’ approaches to care.

“I think we actually need to live our values. We espouse this vision and these values, but we don’t actually practice them sometimes.” (Nurse 1)

Many of the healthcare providers suggested the culture of the organisation and historic hierarchy impacts how the systems, support services and structures are implemented.

“¨at the end of the day, culture is quite difficult to change, and there are just some people who won’t change.” (Medical professional 1)

Parents expressed their gratitude for the services and the care they received through the public health system for their children with chronic and complex conditions. Most parents acknowledged resource restrictions, and the volume of other patients their providers are likely to be treating at the same time as impacting on their, and their child’s, experience.

## Discussion

To our knowledge, this is the first study to incorporate the perspectives of three groups (children and young people with chronic disease, their parents, and healthcare providers) in describing experiences and perceptions of integrated care using an IPA approach. The main finding of this study was that despite these families having access to a care coordination program, the lack of ‘agency and empowerment’ and fragmented and siloed ‘systems’ generally impacted children and young people, their parents’, and healthcare providers’, lived experience of care. The participants described that their experience of care was not consistently oriented around the needs of children, young people and families, nor was it designed and delivered in partnership with them. Our findings suggest that if healthcare providers wish to improve the experiences of their patients, change must occur at multiple levels: interpersonal communication, organisational culture and at a broader system level.

Findings of this study support previous qualitative and quantitative research conducted with healthcare providers, parents and patients (both adult and paediatric), which found that the hierarchical medical model of care is still dominant when providing care [17,21]. Participants in this study highlighted the lack of shared decision making led them to have negative experiences of care and agency [17,21]. Parents and healthcare providers both described the need to respect the expertise they each bring to the care of the child and young person with complex healthcare needs. That is, it needs to be acknowledged by healthcare providers that parents are the experts in their child, and vice versa, that healthcare providers are the clinical experts in their field of medicine or practice. This research identified that this mutual acknowledgement was often inconsistent and impacted on the experiences of care [27]. Previous research showed that healthcare providers who were asked to complete teamwork activities in a laboratory setting did so with no hierarchical, stereotypical and ‘tribal’ behaviours that are often observed in clinical practice [23]. This suggests that organisational workplace culture and structure fosters certain behaviours, rather than the characteristics of the individuals who become healthcare providers [23,28]. Consistent with healthcare providers in the current study, previous research reported that trust and respect between, and appreciation for, the diversity of what different healthcare providers can provide for families are required when addressing fragmentation of care systems [13,29].

From the perspective of children and young people, this study demonstrated that despite previous engagement with a service or healthcare provider, children with chronic conditions may continue to feel unfamiliarity and fear at the thought of going to hospital as a result of having different healthcare providers and being in different environments [18,30]. Allowing children to participate in decision-making about their care builds a sense of control and self-determination [30]. Furthermore, participating in decision making can build awareness of and agency in their own healthcare needs [17,18,20,30]. Healthcare providers acknowledged that parents and carers should be engaged in decision making, however there was not a specific reflection on empowering the children and young people themselves.

The parents in this research valued the targeted coordination, communication, and support provided by CCP nurses. These benefits of care coordination have been recognised in previous research [5,8]. Healthcare providers discussed the challenges of working with children with multiple healthcare providers, and they found some components of caring for these children easier with a dedicated coordinator, as per previous findings [5,13,29]. In qualitative interviews, parents said that the CCP nurse assisted them with medical advocacy, as they were perceived to be able to reduce implementation (of integrated care) barriers of acceptability, appropriateness and feasibility [31]. Despite having a CCP nurse however, parents in the current study felt there was still a lack of clinical oversight of the children’s medical journey. It was identified that clinical oversight and consolidation of all medical results and investigations by a single medic would be beneficial. This need is currently not well addressed, and parents felt this would make a difference in reducing clinical silos, which would lead to better coordinated clinical processes such as blood tests, surgeries, and other procedures.

These findings demonstrated that although individual healthcare providers can provide positive experiences of care, organisational structure and governance mechanisms need to ensure that opportunities for collaboration and partnership with parents, and children and young people are encouraged and supported at all levels. This would promote mutual accountability, respect and genuine collaboration, openness and commitment to shared decision making and quality communication between healthcare providers and with families [27]. Engagement directly with children and young people in a way that is developmentally appropriate and holistic is also key to improving agency and empowerment, which parents expressed would lead to better outcomes. This collaborative approach is key to breaking down the barriers of the traditional hierarchical models of care, to work in a more interprofessional, coordinated and integrated way [13,23,28,32].

### Strengths and limitations

A strength of this study was systematic data collection and analysis using an IPA approach to ensure in-depth exploration of the lived experience of all participants. The focus on a single institution enabled in-depth exploration of the experiences of integrated care relevant to this specific setting and could lead to meaningful organisation and system improvements. The interviews and data analysis were conducted by the same researcher (HJ), who did not have a previous relationship with any of the children or parents, was not involved with the CCP, and had no previous engagement with all but one of the healthcare providers. This contributed to the consistency of questioning and rapport with participants. Parents were present during the interview with their child, as described in the ‘Methods’ section, and as such this may have influenced how the participants responded to the questions. A limitation of the study is that the findings may not be generalisable beyond the tertiary environment studied.

## Conclusion

Care for children and young people with chronic and complex conditions needs to be coordinated, respectful and responsive to the individual and holistic needs of the child and family. Children and young people, their families, and healthcare providers must be partners in care. Healthcare providers must understand the roles and responsibilities of all providers involved in their patients’ care, including recognising families as experts in their child, and vice versa. To do this, quality communication must be recognised as core to care delivery. Healthcare providers must recognise that patients’ and families’ lives are more than their medical condition and consequent healthcare. However, we cannot rely on individual healthcare providers to deliver integrated care consistently in an organisation or system that is not supportive of delivering care this way. The organisational and broader health and wellbeing context in which healthcare delivery occurs impacts how care is delivered to individuals. As such, organisational culture, structure, and governance mechanisms that support this way of working are paramount to the successful implementation of integrated care.

This study contributes to the limited literature base exploring lived experiences of integrated care. The findings encourage the exploration of the three perspectives to support co-design and/or evaluation of health services. Further research informed by theoretical models of culture change to support the implementation of integrated child and family-centred care would be beneficial, as would research into the additional needs of children, young people and families with complex care needs living in rural and remote areas.

## Additional Files

The additional files for this article can be found as follows:

10.5334/ijic.5545.s1 Supplementary file 1. Semi-structured interview guide – sample questions.

10.5334/ijic.5545.s2 Supplementary file 2. Demographics of participating caregivers.

10.5334/ijic.5545.s3 Supplementary file 3. Superordinate themes within children and young people cohort.

10.5334/ijic.5545.s4 Supplementary file 4. Superordinate themes within parent cohort.

10.5334/ijic.5545.s5 Supplementary file 5. Superordinate themes within the healthcare providers cohort.

## References

[1] Altman L, Zurynski Y, Breen C, Hoffman T, Woolfenden S. A qualitative study of health care providers’ perceptions and experiences of working together to care for children with medical complexity (CMC). BMC Health Services Research, 2018; 18(1): 70–70. DOI: 10.1186/s12913-018-2857-8 29386026 PMC5793356

[2] Cohen E, Lacombe-Duncan A, Spalding K, MacInnis J, Nicholas D, Narayanan U, et al. Integrated complex care coordination for children with medical complexity: a mixed-methods evaluation of tertiary care-community collaboration. BMC Health Services Research, 2012; 12: 366 DOI: 10.1186/1472-6963-12-366 23088792 PMC3529108

[3] Cohen E, Friedman JN, Mahant S, Adams S, Jovcevska V, Rosenbaum P. The impact of a complex care clinic in a children’s hospital. Child: Care, Health and Development, 2010; 36(4): 574–82. DOI: 10.1111/j.1365-2214.2009.01069.x 20337643

[4] Graham RJ, MacManus ML, Rodday AM, Weidner RA, Parsons SK. Chronic respiratory failure: Utilization of a pediatric specialty integrated care program. Healthcare, 2017; 5(1–2): 23–28. DOI: 10.1016/j.hjdsi.2016.04.002 28668199

[5] Taylor A, Lizzi M, Marx A, Chilkatowsky M, Trachtenberg SW, Ogle S. Implementing a care coordination program for children with special healthcare needs: partnering with families and providers. Journal for Healthcare Quality, 2013; 35(5): 70–77. DOI: 10.1111/j.1945-1474.2012.00215.x 22913270

[6] Cohen E, Berry JG, Sanders L, Schor EL, Wise PH. Status Complexicus? The Emergence of Pediatric Complex Care. Pediatrics, 2018; 141(Supplement 3): S202 DOI: 10.1542/peds.2017-1284E 29496971

[7] Ling T, Brereton L, Conklin A, Newbould J, Roland M. Barriers and facilitators to integrating care: experiences from the English Integrated Care Pilots. International Journal for Integrated Care, 2012; 12: e129 DOI: 10.5334/ijic.982 PMC3601528 23593044

[8] Nooteboom LA, Kuiper CHZ, Mulder E, Roetman PJ, Eilander J, Vermeiren RRJM. What Do Parents Expect in the 21st Century? A Qualitative Analysis of Integrated Youth Care. International Journal of Integrated Care, 2020; 20(3): 8 DOI: 10.5334/ijic.5419 PMC7442175 32874167

[9] Coller RJ, Nelson BB, Sklansky DJ, Saenz AA, Klitzner TS, Lerner CF, et al. Preventing hospitalizations in children with medical complexity: a systematic review. Pediatrics, 2014; 134(6): e1628–47. DOI: 10.1542/peds.2014-1956 25384492

[10] Productivity Commission. Integrated Care, Shifting the Dial: 5 year Productivity Review, Supporting Paper No. 5 Canberra, Australia: Commonwealth of Australia 2017, [cited 2020 5 Oct]. Available from: https://www.pc.gov.au/inquiries/completed/productivity-review/report/productivity-review-supporting5.pdf

[11] Bodenheimer T, Sinsky C. From Triple to Quadruple Aim: Care of the Patient Requires Care of the Provider. The Annals of Family Medicine, 2014; 12(6): 573–576. DOI: 10.1370/afm.1713 25384822 PMC4226781

[12] Kodner D. All Together Now: A Conceptual Exploration of Integrated Care. Healthcare Quarterly, 2009; 13(Sp): 6–15. DOI: 10.12927/hcq.2009.21091 20057243

[13] Nooteboom LA, Mulder EA, Kuiper CHZ, Colins OF, Vermeiren RJM. Towards Integrated Youth Care: A Systematic Review of Facilitators and Barriers for Professionals. Administration and Policy in Mental Health and Mental Health Services Research; 2020 DOI: 10.1007/s10488-020-01049-8 PMC7803720 32424453

[14] Ziniel SI, Rosenberg HN, Bach AM, Singer SJ, Antonelli RC. Validation of a Parent-Reported Experience Measure of Integrated Care. Pediatrics, 2016; 138(6). DOI: 10.1542/peds.2016-0676 27940672

[15] Queensland Health. Integrated Care Strategy 2018–2022 Brisbane, Queensland: Queensland Health 2018 [cited 2020 5 Oct]. Available from: https://www.childrens.health.qld.gov.au/wp-content/uploads/PDF/our-strategies/integrated-care-strategy-2018.pdf.

[16] Greenfield G, Ignatowicz AM, Belsi A, Pappas, Y, Car J, Majeed A, et al. Wake up, wake up! It’s me! It’s my life! patient narratives on person-centeredness in the integrated care context: a qualitative study. BMC Health Services Research, 2014; 14: 619 DOI: 10.1186/s12913-014-0619-9 25471663 PMC4261575

[17] Ocloo J, Goodrich J, Tanaka H, Birchall-Searle J, Dawson D, Farr M. The importance of power, context and agency in improving patient experience through a patient and family centred care approach. Health Research Policy and Systems, 2020; 18(1): 10 DOI: 10.1186/s12961-019-0487-1 31973712 PMC6979369

[18] Ford K. ‘I didn’t really like it, but it sounded exciting’: Admission to hospital for surgery from the perspectives of children. Journal of Child Health Care, 2011; 15(4): 250–260. DOI: 10.1177/1367493511420185 22089702

[19] Clark H, Coll-Seck AM, Banerjee A, Peterson S, Dalgish SL, Ameragunga S, et al. A future for the world’s children? A WHO–UNICEF–Lancet Commission. The Lancet, 2020; 395(10224): 605–658. DOI: 10.1016/S0140-6736(19)32540-1 32085821

[20] Battrick C, Glasper EA. The views of children and their families on being in hospital. British Journal of Nursing, 2004; 13(6): 328–336. DOI: 10.12968/bjon.2004.13.6.12529 15126966

[21] Arcuri GG, McMullan AE, Murray AE, Silver LK, Bergthorson M, Dahan-Oliel N, et al. Perceptions of family-centred services in a paediatric rehabilitation programme: strengths and complexities from multiple stakeholders. Child: Care, Health and Development, 2016; 42(2): 195–202. DOI: 10.1111/cch.12308 26647743

[22] De S, Tong A, Isaacs D, Craig JC. Parental perspectives on evaluation and management of fever in young infants: an interview study. Archives of Disease in Childhood, 2014; 99(8): 717 DOI: 10.1136/archdischild-2013-305736 24849214

[23] Braithwaite J, Clay-Williams R, Vecellio E, Marks D, Hooper T, Westbrook M, et al. The basis of clinical tribalism, hierarchy and stereotyping: a laboratory-controlled teamwork experiment. BMJ Open, 2016; 6(7): e012467 DOI: 10.1136/bmjopen-2016-012467 PMC4985874 27473955

[24] Smith, JA, Flowers P, Larkin M. Interpretive Phenomenological Analysis: Theory, Method and Research London, England: Sage Publications Ltd.; 2009.

[25] O’Brien BC, Harris IB, Beckman TJ, Reed DA, Cook DA. Standards for reporting qualitative research: a synthesis of recommendations. Academic Medicine, 2014; 89(9): 1245–1251. DOI: 10.1097/ACM.0000000000000388 24979285

[26] Cypress BS. Rigor or Reliability and Validity in Qualitative Research: Perspectives, Strategies, Reconceptualization, and Recommendations. Dimensions of Critical Care Nursing, 2017; 36(4). DOI: 10.1097/DCC.0000000000000253 28570380

[27] Nimmon L, Stenfors-Hayes T. The “Handling” of power in the physician-patient encounter: perceptions from experienced physicians. BMC Medical Education, 2016; 16: 114–114. DOI: 10.1186/s12909-016-0634-0 27091146 PMC4835893

[28] Davies H, Nutley S, Mannion R. Organisational culture and quality of health care. Quality in Health Care, 2000; 9(2): 111 DOI: 10.1136/qhc.9.2.111 11067249 PMC1743521

[29] Nooteboom LA, van den Driesschen SI, Kuiper CHQ, Vermeiren RRJM, Mulder EA. An integrated approach to meet the needs of high-vulnerable families: a qualitative study on integrated care from a professional perspective. Child and Adolescent Psychiatry and Mental Health, 2020; 14(1): 18 DOI: 10.1186/s13034-020-00321-x 32411295 PMC7211334

[30] Coyne I. Children’s experiences of hospitalization. Journal of Child Health Care, 2006; 10(4): 326–336. DOI: 10.1177/1367493506067884 17101624

[31] Proctor E, Silmere H, Raghavan R, Hovmand P, Aarons G, Bunger A, et al. Outcomes for implementation research: conceptual distinctions, measurement challenges, and research agenda. Administration and Policy in Mental Health, 2011; 38(2): 65–76. DOI: 10.1007/s10488-010-0319-7 20957426 PMC3068522

[32] Kassianos AP, Ignatowicz A, Greenfield G, Majeed A, Car J, Pappas Y. “Partners rather than just providers…”: A qualitative study on health care professionals’ views on implementation of multidisciplinary group meetings in the North West London Integrated Care Pilot. International Journal of Integrated Care, 2015; 15: e032–e032. DOI: 10.5334/ijic.2019 26351410 PMC4560079

