# Supplementary material for: Understanding Young People and Their Care Providers’ Perceptions and Experiences of Integrated Care Within a Tertiary Paediatric Hospital Setting, Using Interpretive Phenomenological Analysis
Source: Int J Integr Care. 2020 Oct 27;20(4):7. doi: 10.5334/ijic.5545 (PMC7597574; doi:10.5334/ijic.5545)
Supplement: Supplementary file 1. — Semi-structured interview guide – sample questions. [file ijic-20-4-5545-s1.pdf]

Supplementary file 1. Semi-structured interview guide – sample questions

Cohort	Sample questions
Children and young people	• Can you please tell me about why you come to hospital?<br>o How does it feel coming to the hospital?<br>• How would you describe the way your doctors talk to you?<br>• Do you get to choose what happens here at the hospital?<br>• What other places to you go for your health care?<br>• Do you like school? What do you like at school?<br>• What don't you like at school?<br>• What do you think could make you feel better?<br>• What would make your experiences here better?<br>• Is there anything else you would like to talk to me about?
Parents	• Can you please describe how much you understand your child's condition and care/treatment plans?<br>• Can you please describe what your child's CCP nurse does for you and your child?<br>• How would you describe your involvement in decision making for your child?<br>• How would you describe the way the other health providers (doctors, allied health, nurses) treat your child?<br>• How would you describe the way the other health providers (doctors, allied health, nurses) treat you as a parent/carer?<br>• Do you believe that you are fully informed about your child's care plans and treatment, as much as you want to be?<br>• Do you think all of your child's health providers speak to each other about your child?<br>• How much attention do the health providers pay to you as the parent and carer of your child?<br>• Do they ask you about your family and home everyday life?<br>• Is there anything else you would like to talk to me about?
Healthcare providers	• Can you please describe what you believe integrated care means?<br>• What is your own experience of working in an integrated way?<br>• Can you please describe what you know about the Connected Care program and its benefits to patients?<br>• How well do you think you take into account the needs of the whole family, not just the child you are treating?<br>• When providing care to these children with complex conditions, do you talk to other professionals?<br>• How do you think the system you are working in could be improved for the care of children with complex and chronic conditions?<br>• Is there anything in particular that prevents you from doing the job you personally would like to do?<br>• Is there anything else you would like to talk to me about?
