# Supplementary material for: Understanding Young People and Their Care Providers’ Perceptions and Experiences of Integrated Care Within a Tertiary Paediatric Hospital Setting, Using Interpretive Phenomenological Analysis
Source: Int J Integr Care. 2020 Oct 27;20(4):7. doi: 10.5334/ijic.5545 (PMC7597574; doi:10.5334/ijic.5545)
Supplement: Supplementary file 2. — Demographics of participating caregivers. [file ijic-20-4-5545-s2.pdf]

Supplementary file 2. Demographics of participating caregivers

Variable	Number (%)
**Marital status**	
Two parent household	6 (100%)
**Total number of dependents in household**	
One child	1 (17%)
Two children	3 (50%)
Three children	2 (33%)
**Socioeconomic Index for Areas (SEIFA), Index of Relative Socio-economic Advantage and Disadvantage (IRSAD) status[a]**	
10	2 (33%)
6	1 (17%)
4	1 (17%)
2	1 (17%)
1	1 (17%)
**Working parents**	
One parent	2 (33%)
Two parents	4 (67%)
**Carer**	
Mother (full-time carer)	2 (33%)
Mother (carer + works/studies part-time)	4 (67%)
**Approximate time with Connected Care Program[b] at time of interview (self-reported)**	
< 1 year	2 (33%)
1-2 years	2 (33%)
4+ years	1 (17%)
Unsure	1 (17%)
**Approximate number of clinical teams at time of interview (self-reported)**	
4	2 (33%)
5-10	2 (33%)
>10	1 (17%)
Unsure	1 (17%)

[a] Score 1-10 (1 = area of relatively greater disadvantage and a lack of advantage in general; 10 = lack of disadvantage and greater advantage in general)

[b] Established in 2013
