# Supplementary material for: Understanding Young People and Their Care Providers’ Perceptions and Experiences of Integrated Care Within a Tertiary Paediatric Hospital Setting, Using Interpretive Phenomenological Analysis
Source: Int J Integr Care. 2020 Oct 27;20(4):7. doi: 10.5334/ijic.5545 (PMC7597574; doi:10.5334/ijic.5545)
Supplement: Supplementary file 3. — Superordinate themes within children and young people cohort. [file ijic-20-4-5545-s3.pdf]

Superordinate theme	Meaning	Example quote	Child 1	Child 2	Child 3	Child 4	Young person 1	Young person 2
Knowledge and understanding	Reason for attending hospital, of their condition	"Interviewer: What do you do when you come here?<br>Interviewee: I don't know." *(Child 3, age 7 years)*	X	X	X		X	
Engagement	Shared decision-making, choice, trust it staff, kindness, developmentally appropriate involvement in care, feeling safe	"Interviewee: They give her [mother] lots of information..<br>Interviewer: Mm-hm. Do they give you information?<br>Interviewee: No.<br>Interviewer: Just to your mum?<br>Interviewee: Yeah." *(Child 2, 8 years)*	X	X	X	X	X	X
Comfort and safety	Valuing family and home time; doesn't like to come; anxious and scared; likes comforts of home and family; trust and support; parents support.	"Interviewer: Where's your favourite place to go?<br>Interviewee 1: Home." *(Child 1, 10 years)*	X	X	X	X	X	X

Supplementary file 3. Superordinate themes within children and young people cohort
