# Supplementary material for: Understanding Young People and Their Care Providers’ Perceptions and Experiences of Integrated Care Within a Tertiary Paediatric Hospital Setting, Using Interpretive Phenomenological Analysis
Source: Int J Integr Care. 2020 Oct 27;20(4):7. doi: 10.5334/ijic.5545 (PMC7597574; doi:10.5334/ijic.5545)
Supplement: Supplementary file 4. — Superordinate themes within parent cohort. [file ijic-20-4-5545-s4.pdf]

Supplementary file 4. Superordinate themes within parent cohort

| **Superordinate theme** | **Meaning** | **Example quotes** | **Parent 1** | **Parent 2** | **Parent 3** | **Parent 4** | **Parent 5** | **Parent 6** |
|---|---|---|---|---|---|---|---|---|
| Person-centred care | partnership, holistic care tailored to the needs of child and family | "I think what else I like is [specialty] doctors do a holistic approach towards [de identified]'s healthcare and they ask about the other areas and about her in general." *(Parent 3)* | X | X | X | X | X | X |
| Communication and engagement | timely, genuine, consistent, open, facilitates shared decision making | "When someone who is actually talking to [de-identified like a person and they are talking to her as if they just want to help, rather than someone who is just trying to get her out the door next, that is asking the question because they're in a children's hospital and they should say, hi how are you [de-identified] - and they're just trying to tick that box of yes I've said hello to the patient and I've spoken to the child directly, now I need to talk to the parents and ignore the child. So, they're just trying to get their job done and see the next person which we understand, public system, it happens. But it's the genuineness behind it, I guess, is lacking a lot of the time." *(Parent 2)* | X | X | X | | | |
| Power imbalance | hierarchy between healthcare providers and parents and children and young people | "That can be confronting - even when you have a longstanding and even when you have a very good relationship - there is a power imbalance and it takes a very good doctor having a good day to be cognisant of that and to work as a team and plan and discuss and debate, even. Sometimes it's debate - like work through stuff when it's complex." *(Parent 4)* | | X | X | X | | X |
| Inconsistency and inequity | of care, processes, empathy, support, accountability and respect | "How is there such a big difference in the level of care, just because it's a different person? When - does the hospital meant to regulate that? Is it - how do they know that the level of | X | X | X | X | X | X |

| | | | | | | | | |
|---|---|---|---|---|---|---|---|---|
| | | care is so different between different doctors in the same specialty?” *(Parent 2)* | | | | | | |
| Systems and supports | scheduling, care coordination, advocacy, efficiency | “They blame it on the system, but it happens so often.  I don't know what I should do, because I didn't know, I don't know if I can call people up or who to call.  So, it's been really nice to have Connected Care to go, hey, just wondering if she has an appointment coming.” *(Parent 3)* | X | X | X | | X | X |
