# Supplementary material for: Understanding Young People and Their Care Providers’ Perceptions and Experiences of Integrated Care Within a Tertiary Paediatric Hospital Setting, Using Interpretive Phenomenological Analysis
Source: Int J Integr Care. 2020 Oct 27;20(4):7. doi: 10.5334/ijic.5545 (PMC7597574; doi:10.5334/ijic.5545)
Supplement: Supplementary file 5. — Superordinate themes within the healthcare providers cohort. [file ijic-20-4-5545-s5.pdf]

Supplementary file 5. Superordinate themes within the healthcare providers cohort

Superordinate theme	Meaning	Example quotes	Nurse 1	Nurse 2	Allied health professional 1	Allied health professional 2	Medical professional 1	Medical professional 2
Traditional models of care	medical model, sub-specialisation, power imbalance, hierarchy, siloed care, reactive	"I think it's also the way the clinical specialties are - it's the culture and it's the way they're set up. A lot of it is cultural and it's steeped in, that's the way that it's always been done." *(Nurse 1)*	X	X	X	X	X	X
Embracing child and family centred care	respect, responsive to child and family's needs, holistic care, empowering, partnering, building health literacy	"..the family are placed front and centre and their needs are also placed front and centre. As opposed to being a splintered system of care where the access care, you know, independent from one team to the next with very little communication." *(Allied health professional 1)*	X	X	X	X	X	X
Care coordination	coordinating services, single point of contact, advocacy and accountability	"..one person to contact all the time who can help coordinate some of their appointments, but not only that, can actually offer them some further detail and information around understanding the medical jargon that doctors use, the need for certain tests and appointments but also bring a whole big support about when they are actually at	X	X	X	X	X	

		home and living in their own community that they can actually tap into other resources there as well." *(Allied health professional 2)*						
Communication	between healthcare providers and with children, young people and families, timely, open, consistent, respectful, management of expectations	"I think communication still is a barrier. I think people do it the best they can but people are time poor. So to make sure you have communicated with every team and every member of the team, that's important, at any given time on the days that they're there and when a patient is in is tricky and requires a lot of forward planning and work and chiming in with procedures that are occurring at any given time." *(Allied health professional 1)*		X	X		X	X
Interprofessional practice	role clarity, team functioning, collaborative (non-hierarchical) leadership, communication	"I do think there's sometimes misunderstanding about individual roles." *(Medical professional 1)*	X	X	X	X	X	X
Organisational influences	scheduling, staffing, resourcing, time, rosters, values and culture, environmental impacts, systems, procedures and processes, leadership	"I think quite simply it's actually booking services in the database. It all comes down to IT and technology; how good is the software that you've got and how efficient is it the way we run it? The delineation and the	X	X	X	X	X	

		separating between bookings and different teams and stuff, not being able to streamline things makes it difficult.” *(Allied health professional 2)*						
